# Investigation of Antireflection Nb_2_O_5_ Thin Films by the Sputtering Method under Different Deposition Parameters

**DOI:** 10.3390/mi7090151

**Published:** 2016-09-01

**Authors:** Kun-Neng Chen, Chao-Ming Hsu, Jing Liu, Yu-Chen Liou, Cheng-Fu Yang

**Affiliations:** 1 Department of Electrical Engineering, Kun-Shan University, Tainan 710, Taiwan; knchen@mail.ksu.edu.tw; 2 Department of Mechanical Engineering, National Kaohsiung University of Applied Science, Kaohsiung 807, Taiwan; jammy@kuas.edu.tw; 3 School of Information Engineering, Jimei University, Xiamen 361021, China; jingliu@jmu.edu.cn; 4 Department of Chemical and Materials Engineering, National University of Kaohsiung, Kaohsiung 811, Taiwan; y36373839@gmail.com

**Keywords:** Nb_2_O_5_ thin films, anti-reflection, deposition temperature, transmittance, optical band gap

## Abstract

In this study, Nb_2_O_5_ ceramic was used as the target to deposit the Nb_2_O_5_ thin films on glass substrates with the radio frequency (RF) magnetron sputtering method. Different deposition temperatures and O_2_ ratios were used as parameters to investigate the optical properties of Nb_2_O_5_ thin films. The deposition parameters were a pressure of 5 × 10^−3^ Torr, a deposition power of 100 W, a deposition time of 30 min, an O_2_ ratio (O_2_/(O_2_ + Ar), in sccm) of 10% and 20%, and deposition temperatures of room temperature (RT), 200, 300 and 400 °C, respectively. We found that even if the deposition temperature was 400 °C, the deposited Nb_2_O_5_ thin films revealed an amorphous phase and no crystallization phase was observed. The optical properties of transmittance of Nb_2_O_5_ thin films deposited on glass substrates were determined by using a ultraviolet-visible (UV-vis) spectrophotometer (transmittance) and reflectance spectra transmittance (reflectance, refractive index, and extinction coefficient) in the light wavelength range of 250–1000 nm. When the O_2_ ratio was 10% and the deposition temperature increased from RT to 200 °C, the red-shift was clearly observed in the transmittance curve and the transmission ratio had no apparent change with the increasing deposition temperature. When the O_2_ ratio was 20%, the red-shift was not observed in the transmittance curve and the transmission ratio apparently decreased with the increasing deposition temperature. The variations in the optical band gap (*E_g_*) values of Nb_2_O_5_ thin films were evaluated from the Tauc plot by using the quantity *hν* (the photon energy) on the abscissa and the quantity (α*hν*)*^r^* on the ordinate, where α is the optical absorption coefficient, *c* is the constant for direct transition, *h* is Planck’s constant, *ν* is the frequency of the incident photon, and the exponent *r* denotes the nature of the transition. As the O_2_ ratio of 10% or 20% was used as the deposition atmosphere, the measured *E_g_* values decreased with the increase of the deposition temperature. The reflectance ratio, extinction coefficient, and refractive index curves of Nb_2_O_5_ thin films were also investigated in this study. We would show that those results were influenced by the deposition temperature and O_2_ ratio.

## 1. Introduction

Different metal-oxide thin films are increasingly applied for a wide range of optical and microelectronic applications. In those materials, the most stable form of niobium oxides is Nb_2_O_5_ and it is very attractive for electronic and optical applications. Nb_2_O_5_, along with other oxides of metals from the Vth group, have also been investigated as possible candidates for thin film catalysts and corrosion barrier coatings [1,2]. In the case of optical applications, Nb_2_O_5_-based thin films are often used as high index and low loss materials; for example, they can be used as optical waveguides [3,4], interference filters, antireflective or antireflection (AR) coatings or electroluminescent devices [5,6,7]. An AR coating is a type of optical coating applied to the surface of lenses and other optical elements to reduce reflection. For example, bare silicon–based solar cells have a high surface reflection of over 30%. AR coatings are applied to reduce surface reflection and maximize the devices’ efficiency for the solar cells manufactured on glass and silicon substrates [8]. The simplest interference antireflection (AR) coating can be achieved by using a single quarter-wave layer of oxide thin films whose refractive index is chosen as the square root of the substrate’s refractive index. The single-layer AR coating consists of a dielectric thin film. As the thin films have a specially chosen thickness, the interference effects in the AR coating will cause the wave to be reflected from the top surface of the coating layers to be out of phase with the wave reflected from the surfaces of the semiconductor [9]. For that, the thin films can have an effect of zero reflectance at the center light wavelength which can decrease reflectance for light wavelengths in a broad band around the center. As the multi-layer AR coating thin films are used, the alternating layers of a low-index material (such as SiO_2_) and a higher-index material (such as ZrO_2_) are possible to obtain reflectivity as low as 0.1% at a special or single wavelength [10,11].

Nb_2_O_5_ is one of the useful optical thin-film materials because of its desirable properties, including stability in air and water and resistance to acids and bases. Also, the Nb_2_O_5_ thin films have the properties of high refractive index, low extinction coefficient, and high transparent ratio in the UV-vis-NIR (ultraviolet-visible near-infrared) region [12,13]. The properties of Nb_2_O_5_ thin films closely rely on the sputtered materials, deposition techniques, deposition parameters, and the thicknesses of thin films. For that, many different methods are investigated to deposit Nb_2_O_5_ thin films. For example, Nb_2_O_5_ thin films can be deposited on variety of substrates using the sol-gel dip coating technique [14], and the deposited Nb_2_O_5_ thin films revealed an amorphous phase [1,15,16]. Agarwal and Reddy found that after annealing Nb_2_O_5_ thin films under controlled ambience at 600 °C for 5 h, the thin films deposited on NaCl substrates crystallized into a stable monoclinic phase and those deposited on single-crystal Si substrates crystallized into a hexagonal phase [13]. The optical band gap of Nb_2_O_5_ thin films increased from 4.35 eV when the films were in an amorphous state to 4.87 eV on crystallization [13]. Lazarova et al. obtained Nb_2_O_5_ thin films by mixing NbCl_5_ with ethanol to form the spin-coating precursor. The mixing material was spin-coated on glass and silicon substrates and then they were annealed at different temperatures to obtain the Nb_2_O_5_ thin films [17]. Nb_2_O_5_ thin films could also be formed by annealing the sputter-deposited Nb thin films under a pure Ar flow plasma and annealing them in a quartz tube at 850 °C. Jose et al. observed vibrational modes including longitudinal optical, transverse optical, and triply degenerate modes, and found the direct optical band gap to be ~3.49 eV [18].

Sputtering is a simple method to deposit Nb_2_O_5_ thin films with a controllable thickness [19]. In the sputtering method, the properties of the Nb_2_O_5_ thin films depend on the deposition parameters, such as the deposition temperature [20], reactive gas flow or pressure. In this paper, Nb_2_O_5_ thin films were deposited on glass substrates by using the sputtering method. The effects of the deposition temperature and oxygen concentration (10% and 20%) in the deposition atmosphere on the properties of Nb_2_O_5_ thin films were investigated by X-ray diffraction (XRD) patterns and field emission scanning electron microscopy (FESEM), respectively. In this paper we found two important novelties. The first novelty in this paper is that the Nb_2_O_5_ thin films deposited in pure argon atmosphere revealing an un-transparent result. The second novelty is that the O_2_ flow rate has a large effect on the properties of Nb_2_O_5_ thin films; only a few studies focused on the effect of the O_2_ flow rate on that topic. In the past, the Nb_2_O_5_ thin films had been investigated by different methods, for example by the microwave-assisted reactive magnetron sputtering process [6], by the glancing angle deposition process [12], by the sol-gel dip coating technique [13], by the sol-gel and evaporation-induced self-assembly method [14], by radio frequency (RF) magnetron sputtering [16], and by a low frequency reactive magnetron sputtering system [19], respectively. In the past, as the physical plasma method was used to deposit the Nb_2_O_5_ thin films, no reports were focused on the effect of the O_2_ flow rate in the (O_2_ + Ar) mixing atmosphere on the properties’ extinction coefficient, optical band gap (*E_g_*) values, and refractive index. Coskuna et al. used the RF magnetron sputtering method to deposit the Nb_2_O_5_ thin films [16], and they investigated the effect of the deposition temperature but they did not investigate the effect of the O_2_ flow rate on the properties of Nb_2_O_5_ thin films. Mazur et al. used the microwave-assisted reactive magnetron sputtering process [6] and Lai et al. used the low frequency reactive magnetron sputtering system [19] to deposit the Nb_2_O_5_ thin films by using a metallic Nb target in the O_2_ + Ar mixing atmosphere, and they also did not investigate the effect of the O_2_ flow rate on the properties of Nb_2_O_5_ thin films.

RF magnetron sputtering is a well-established high-rate vacuum coating method for the deposition of industrial thin film materials and the thickness of deposited thin films can be well controlled by controlling the deposition parameters. For that, we used RF magnetron sputtering and the Nb_2_O_5_ ceramic target to deposit the Nb_2_O_5_ thin films. We found that the O_2_ flow rate had a large effect on the transmittance and reflectance of Nb_2_O_5_ thin films. Additionally, optical properties of Nb_2_O_5_ thin films, such as transmittance, reflectance, and reactance, were determined by using a spectrophotometer and elliposmeter. The second novelty is that we discussed different O_2_ flow rates in the (O_2_ + Ar) mixing atmosphere to investigate its effect on the properties of Nb_2_O_5_ thin films, especially on the extinction coefficient, optical band gap (*E_g_*) values, and refractive index. For example, the refractive index of Nb_2_O_5_ thin films increased with the increase of the O_2_ flow rate in the (O_2_ + Ar) mixing atmosphere. The results in this study show that the Nb_2_O_5_ antireflective layer with protective properties can be proposed for possible use in silicon solar cells.

## 2. Experimental Section

At the first, Nb_2_O_5_ powder was mixed with acetone, dried, and ground, and mixed with polyvinyl alcohol (PVA) as binder. After being debindered, Nb_2_O_5_ ceramic was sintered at 1400 °C for 2 h in air. The crystalline phases were analyzed by using X-ray diffraction (XRD) patterns to find the crystallization of Nb_2_O_5_ ceramic, and only the Nb_2_O_5_ phase was observed in the sintered ceramic. Glass substrates (Corning 1737, Coring, New York, NY, USA) with an area of 2 × 2 cm^2^ were cleaned ultrasonically with isopropyl alcohol (IPA) and deionized (DI) water and then dried under a blown nitrogen gas. Deposition power of Nb_2_O_5_ thin films was 100 W, deposition time was 30 min, and the deposition temperatures were room temperature (RT), 200, 300 and 400 °C, respectively. In this study, the radio frequency (RF) magnetron sputtering (SYSKEY, Hsinchu, Taiwan) was used as equipment to deposit the Nb_2_O_5_ thin films. The base pressure of sputtering chamber was below 5 × 10^−6^ Torr, the deposition time and power were 30 min and 100 W, the working pressure was maintained at 3 × 10^−3^ Torr in O_2_ 5 sccm-Ar 45 sccm (O_2_ ratio of 10%) and in O_2_ 10 sccm-Ar 40 sccm (O_2_ ratio of 20%) ambient. Thickness and surface morphology of Nb_2_O_5_ thin films were measured using a field emission scanning electron microscopy (FESEM), the roughness (or flatness) was measured using atomic force microscopy (AFM), and their crystalline structures were measured using X-ray diffraction (XRD) patterns with Cu Kα radiation (λ = 1.5418 Å). The optical transmission spectrum was recorded using a Hitachi U-3300 ultraviolet–visible (UV-vis) spectrophotometer in the 250–1000 nm light wavelength range and the reflectance spectra of the films measured at normal light incidence by UV-vis-NIR spectrophotometer Cary 05E (Varian Australia Pty. Ltd., Mulgrave, Australia) using non-linear curve fitting method, respectively. Refractive index (RI) is a complex number comprising a real refractive index and an imaginary part: the absorption (or extinction) coefficient. Therefore, the well-established technique of ellipsometry can determine both the real refractive index (*n*) and extinction coefficient (*k*). The optical properties (*n* and *k*) along with the thickness of Nb_2_O_5_ thin films were determined from n&k Analyzer 1280 (n&k Technology, San Jose, CA, USA). At the first, the baseline sample was put on the n&k Analyzer 1280, after that the measured samples were put on the n&k Analyzer 1280 and the software would calculate the *n* and *k* value.

## 3. Results and Discussion

**Figure 1:**
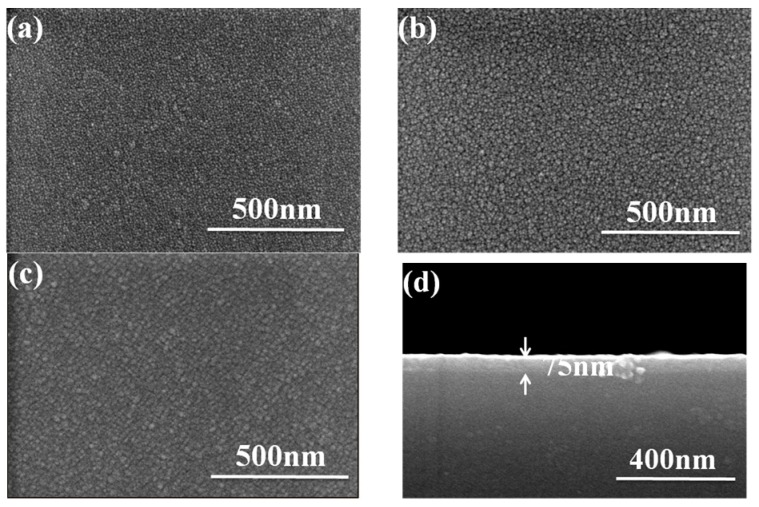
Surface morphology and cross observations of Nb_2_O_5_ thin film deposited at (**a**) room temperature (RT), (**b**) 300 °C, and (**c**) 400 °C, respectively. (**d**) Cross-section observation of Nb_2_O_5_ thin films, the deposition temperature was 200 °C and the O_2_ ratio was 10%, respectively.

Figure 1 shows the surface and cross-section observations of Nb_2_O_5_ thin films with various deposition temperatures and the O_2_ ratio was 10%. As the results in Figure 1 show, high resolution SEM reveals that when the deposition temperature was RT, there was a densified surface morphology, consisting of nanocrystalline particles growing in a random orientation. Further increasing the deposition temperature to 300 and 400 °C caused the Nb_2_O_5_ thin films to have larger nanocrystalline grains. Even when the O_2_ ratio was changed to 20%, the variations in the surface morphology of the Nb_2_O_5_ thin films had similar results, and the sizes of the nanocrystalline grains increased (not shown here). The root mean square (RMS) surface roughness of the Nb_2_O_5_ thin films was measured to be 0.30 nm by AFM. When the deposition temperature was RT, 200, 300 and 400 °C, the measured values were 2.56, 3.46, 4.39 and 5.25 nm when the O_2_ ratio was 10% and the measured values were 2.44, 3.32, 4.24 and 5.08 nm when the O_2_ ratio was 20%, respectively. Apparently, the roughness increased as the deposition temperature was raised. Apparently, the results show that the roughness of the Nb_2_O_5_ thin films increased with the increasing deposition temperature. The results in Figure 1 have also revealed an important result: that the nanocrystalline grains had uniform particle sizes. The average crystallite sizes of Nb_2_O_5_ thin films can be calculated using the following equation:

*G* = −2.9542 + 1.4427ln(*N*) (1)

where *G* is the number of grains per unit area at a particular magnification, *N* is the number of grains/mm^2^. The average crystallite sizes were about 12.3, 16.5, 22.7 and 34.5 nm when the deposition temperatures were RT (Figure 1a), 200 °C (not shown here), 300 °C (Figure 1b), and 400 °C (Figure 1c), respectively. When the O_2_ ratio was 20%, the average crystallite sizes were about 11.2, 15.1, 21.4 and 32.5 nm when the deposition temperatures were RT, 200 °C, 300 °C, and 400 °C, respectively (not shown here).

The average crystallite sizes and the deviation of the crystallite sizes of Nb_2_O_5_ thin films are shown in Table 1. We believe that the higher deposition temperature will cause the Nb_2_O_5_ molecules to have a large activation energy and then the Nb_2_O_5_ thin films have a larger particle size and larger roughness. The thickness of Nb_2_O_5_ thin films is also observed and the result is shown in Figure 1d. The thickness was around 75 nm when the deposition temperature was 200 °C and the O_2_ ratio was 10%, respectively. We found that as the deposition temperature was changed from RT to 400 °C and the O_2_ ratio was 10% and 20%, the average thicknesses of Nb_2_O_5_ thin films were in the range of 72.7–77.7 nm and 46.4–47.9 nm, respectively. The average thicknesses and the deviation of the thicknesses of Nb_2_O_5_ thin films are also shown in Table 1. Those results suggest that in this study, the O_2_ ratio has no effect but the deposition temperature has apparent effect on the surface morphology, and the O_2_ ratio has an effect but the deposition temperature has no apparent effect on the thickness, respectively.

**Figure 2:**
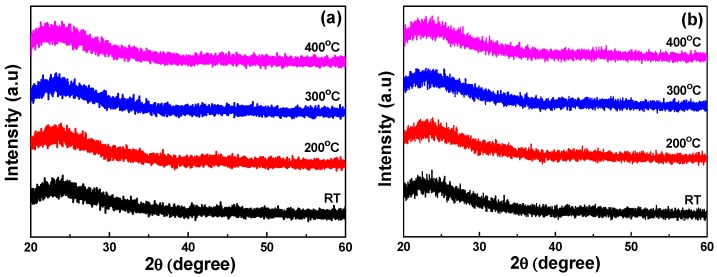
X-ray diffraction (XRD) patterns of Nb_2_O_5_ thin films as a function of deposition temperature and O_2_ ratio. (**a**) O_2_ ratio 10% and (**b**) O_2_ ratio 20%, respectively.

The XRD patterns of Nb_2_O_5_ thin films developed as a function of deposition temperatures are shown in Figure 2a, where the oxygen concentration was 10%. As the different sintering temperatures were used, the differently crystalline phases would be formed in the Nb_2_O_5_ ceramic targets, and the multi-crystal phases were only observed in the Nb_2_O_5_ ceramic targets. Nb_2_O_5_ thin films deposited by the sputtering method in the O_2_/Ar mixed atmosphere would reveal the amorphous phase rather than the polycrystal phase since no characteristic peaks were observed in the XRD patterns. The amorphous nature of the deposited thin films can result from the low temperature of the sputtering process. Even when the Nb_2_O_5_ thin films were deposited at 400 °C, the deposition temperature was not high enough to crystallize the Nb_2_O_5_ thin films. Figure 2b shows that when the oxygen concentration was 20% and the deposition temperature was 400 °C, the XRD patterns of Nb_2_O_5_ thin films also revealed the amorphous phase.

**Figure 3:**
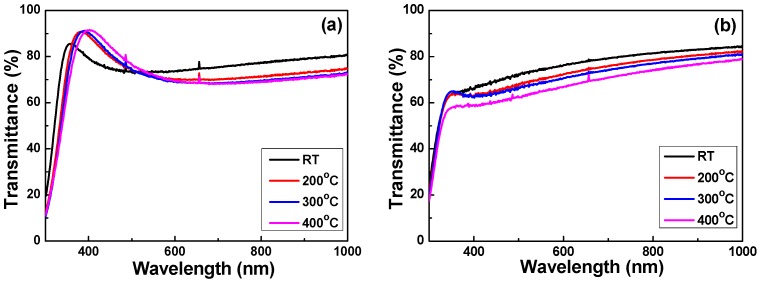
Measured transmittance of Nb_2_O_5_ thin films as a function of deposition temperature and O_2_ ratio. (**a**) O_2_ ratio 10% and (**b**) O_2_ ratio 20%, respectively.

The transmittance of Nb_2_O_5_ thin films under various deposition temperatures and O_2_ ratios is depicted in Figure 3. When the O_2_ ratio was 10%, the average total transmittance of the RT-deposited Nb_2_O_5_ thin films was 74.42%, whereas the Nb_2_O_5_ thin films showed that the average total transmittance was almost unchanged with the increasing deposition temperature in the visible-light wavelength (400–800 nm) region. The average total transmittances of 74.15%, 73.87%, and 75.17% were shown as the deposition temperature was 200, 300 and 400 °C, respectively. The maximum transmittance ratio was 85.73% at 361 nm, 90.78% at 382 nm, 91.06% at 387 nm, and 91.54% at 402 nm, respectively. However, when the O_2_ ratio was 20%, the average total transmittances of the Nb_2_O_5_ thin films had different results. When the deposition temperature was RT, 200, 300 and 400 °C, the average total transmittance was 73.82%, 69.90%, 68.42%, and 64.72%, respectively. Apparently, the average total transmittances of the Nb_2_O_5_ thin films decreased with the increasing deposition temperature in the visible-light wavelength region. The results in Figure 3b also show that the transmittance of the Nb_2_O_5_ thin films increased with the increasing measuring frequency. The red-shift of the maximum transmittance in Nb_2_O_5_ thin films was also apparently observed as the deposition temperature increased. When the same deposition temperature was used, in the transmission spectra of the 20% O_2_-deposited Nb_2_O_5_ thin films, the optical band edge was also shifted to a shorter light wavelength. The optical transmittance properties of Nb_2_O_5_ thin films are listed in Table 2.

**Figure 4:**
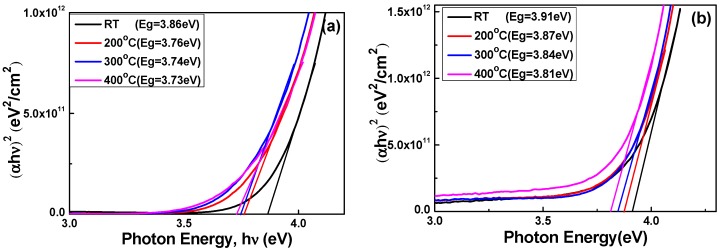
The (α*hv*)^2^ versus *hν* − *E*_g_ plots of Nb_2_O_5_ thin films as a function of deposition temperature and O_2_ ratio. (**a**) O_2_ ratio 10% and (**b**) O_2_ ratio 20%, respectively.

When the oxygen ratio was 10% and the deposition temperature increased from RT to 400 °C, the optical band edges in the transmission spectra of the Nb_2_O_5_ thin films were shifted to a longer light wavelength and a greater sharpness was noticeable in the curves of the absorption edges. Those results mean that as the deposition temperature increases, the absorption edge of the Nb_2_O_5_ thin films is red-shifted. In the past, the determination of the optical band gap (*E*_g_) was often necessary to develop the electronic band structure of a thin film material. Typically, a Tauc plot shows the quantity *hν* (the photon energy) on the abscissa and the quantity (α*hν*)*^r^* on the ordinate, where α is the optical absorption coefficient, *c* is the constant for direct transition, *h* is Planck’s constant, *ν* is the frequency of the incident photon, and the exponent *r* denotes the nature of the transition [21]. When using the Tauc plot method, the *E*_g_ values of thin films can be determined from the absorption edge for the direct interband transition, which can be calculated using the relation in Equation (1) [21]:

(α*hv*)^2^ = *c*(*hν* − *E*_g_) (2)

where α is the optical absorption coefficient, *c* is the constant for direct transition, *h* is Planck’s constant, and *ν* is the frequency of the incident photon [21]. Figure 4 shows the energy band gap of the plasma-treated Nb_2_O_5_ thin films plotted against light wavelengths in the region of 300–1000 nm. The linear dependence of (α*hv*)^2^ on *hν* indicates that Nb_2_O_5_ thin films are a direct transition material [21]. The experimental band-gap width of Nb_2_O_5_ is generally measured in the order of 3.3 to 3.9 eV [22,23]. In accordance with Equation (1), the calculated optical band gap of the Nb_2_O_5_ thin films decreased from 3.86 to 3.73 eV when the O_2_ ratio was 10% and decreased from 3.91 to 3.81 eV when the O_2_ ratio was 20%. As the results in Figure 4a,b were compared, we found that as the same deposition temperature was used to deposit Nb_2_O_5_ thin films, the *E*_g_ value for using the O_2_ ratio of 20% was higher than that for using the O_2_ ratio of 10%.

The optical band gap of amorphous materials depends on their structure and components; thus, it is closely related to the deposition techniques. For a transparent material or thin film, when the light wavelength is equal to 300 nm, the visible light absorbed by the thin films is due to a quantum phenomenon called band edge absorption [24]. In this study, the thicknesses of Nb_2_O_5_ thin films are about 75 nm when the O_2_ ratio is 10% and about 47 nm when the O_2_ ratio is 20%. Because the thicknesses of Nb_2_O_5_ thin films are smaller than 300 nm, the band edge absorption effect will not happen. We believed that the decrease in the band gap with the increased deposition temperature could be attributed to a shift in either the conduction or valence bands leading to narrower energy band gaps. In the past, the red-shift effect could be explained by the Burstein-Moss shift, a shift of the Fermi level into the conduction band, which enhances the optical band gap by the energy, as follows [25,26]:

(3) $$\Delta E_{g}^{\mathrm{BM}}=\frac{\hbar^{2}k_{F}^{2}}{2}\left(\frac{1}{m_{e}}+\frac{1}{m_{h}}\right)=\frac{\hbar^{2}k_{F}^{2}}{2m_{\mathrm{vc}}^{*}}$$

where *k*_F_ stands for the Fermi wave vector, *m*_e_ is the effective mass of electrons in the conduction band, and *m*_h_ is the effective mass of holes in the valence band. The Nb_2_O_5_ thin films are an insulating material as the oxygen is introduced during the deposition process. For that, the Burstein-Moss shift is not the reason that causes the increase in the value of the optical band gap. The formal Nb-oxidation state of Nb_2_O_5_ is +5 and the corresponding electronic configuration of the Nb atoms is [Kr]4d^0^, i.e., all of the d-electrons have been transferred to the O_2p_ band and the Nb_4d_ band is empty [23]. Moreover, Brayner et al. experimentally observed that the energy of the absorption edge is influenced not only by the number of interconnected polyhedrals (size effect) but also by the local coordination as the particle sizes decrease from about 40 to 4.5 nm. They also found that the transmission spectrum shows a significant blue shift of the absorption edge and an increase of the optical band gap from 3.4 to 4.2 eV as the size of the Nb_2_O_5_ particles decreases from about 40 to 4.5 nm. They ascribed this modification to a quantum-size effect [27]. Figure 1 shows that particle sizes of the Nb_2_O_5_ thin films increased with the increasing deposition temperature and Figure 4 shows that the values of the optical band gap decreased with the increasing deposition temperature. We believe that as the deposition temperature increases, the quantum-size effect is the reason that causes the decrease in the optical band gap values of Nb_2_O_5_ thin films.

**Figure 5:**
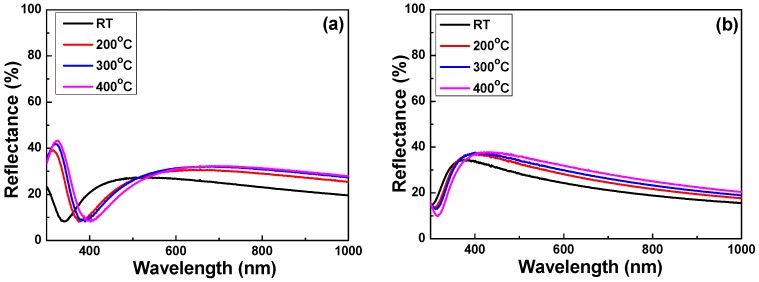
Measured reflectance of Nb_2_O_5_ thin films as a function of deposition temperature and O_2_ ratio. (**a**) O_2_ ratio 10% and (**b**) O_2_ ratio 20%, respectively.

Reflectance is a ratio of the intensity of the incident optical light or other electromagnetic radiation on the surface of a material or a thin film. As usual, the reflectance spectrum can be plotted as a function of the light wavelength. Figure 5 shows the measured reflectance of Nb_2_O_5_ thin films as a function of the deposition temperature and the O_2_ ratio in the light wavelength range of 350–1000 nm. As we know, reflectivity can be defined as the square of the magnitude of the Fresnel reflection coefficient [28], which is the ratio of the reflected electric field to incident on a material or a thin film. If the reflection occurs from a thin layer of materials and the thicker the sample that is used, the effects of the internal reflection can cause the measured reflectance to change as the surface thickness and reflectivity are the limit values of reflectance. As the spectrum in Figure 5a shows, as the O_2_ ratio was 10%, the average reflectances of 26.0%, 26.3%, 26.6%, and 25.3% were shown as the deposition temperature was RT, 200, 300 and 400 °C.

The reflectance first increased as the light wavelength increased and reached a maximum at around 325 nm (except the RT-deposited thin films), then it decreased and reached a minimum at around 400 nm. The minimum reflectance was 8.13% in the 400 °C–deposited Nb_2_O_5_ thin films. As the spectrum in Figure 5b shows, when the O_2_ ratio was 20%, the average reflectances of 26.7%, 30.6%, 32.0%, and 33.7% were shown as the deposition temperature was RT, 200, 300 and 400 °C, respectively. The reflectance first increased as the light wavelength increased and the reflectance reached a maximum when the light wavelength was around 400 nm. When the light wavelength increased from 400 to 1000 nm, the reflectance linearly decreased. The results shown in Figure 3 and Figure 5 suggest that the O_2_ ratio is an important factor that affects the optical properties of Nb_2_O_5_ thin films. The red-shift of the minimum reflectance in the Nb_2_O_5_ thin films was also apparently observed as the deposition temperature increased. The optical reflectance properties of Nb_2_O_5_ thin films are listed in Table 3.

**Figure 6:**
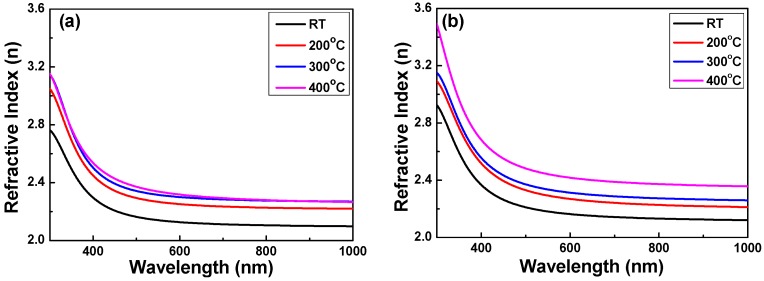
Measured refractive index of Nb_2_O_5_ thin films as a function of deposition temperature and O_2_ ratio. (**a**) O_2_ ratio 10% and (**b**) O_2_ ratio 20%, respectively.

In optics, the refractive index or index of refraction *n* of an optical medium is a dimensionless number that describes how light, or any other radiation, propagates through that medium. It is defined as:

*n* = *c*/*v* (4)

where *c* is the speed of light in a vacuum and *v* is the phase velocity of light in the medium. The refractive index is not a stable value and it will vary with the light wavelength. That is called dispersion and it causes the splitting of white light into its constituent colors into rainbows and chromatic aberration in lenses. However, the light propagation in absorbing materials is very complicated and can be described using a complex-valued refractive index. We can measured the refractive index by using n&k Analyzer 1280 and Figure 6 shows the measured refractive index of Nb_2_O_5_ thin films as a function of the deposition temperature and O_2_ ratio.

No matter whether the O_2_ ratio was 10% or 20%, the refractive index of the Nb_2_O_5_ thin films decreased with the increasing light wavelength. For example, as Figure 6a shows for when O_2_ ratio was 10%, the refractive index decreased from 3.14 to around 2.3 when light wavelength was longer than 450 nm and then the refractive index had a stable value when the light wavelength was longer. Figure 6b (when O_2_ ratio was 20%) also shows that the refractive index at the same light wavelength increased as the deposition temperature was increased from RT to 200 °C and slightly increased as the deposition temperature was further increased. As Figure 6b shows, the variation in the refractive index of Nb_2_O_5_ thin films was similar to that of Nb_2_O_5_ thin films with an O_2_ ratio of 10%. As the results in Figure 6 are compared, we have found that as the same deposition temperature was used, the refractive index of the Nb_2_O_5_ thin films deposited with the O_2_ ratio of 20% was higher than that deposited with the O_2_ ratio of 10%. Those results suggest again that the deposition temperature and O_2_ ratio are two important factors that will affect the properties of Nb_2_O_5_ thin films. Those results suggest that as a higher substrate temperature is used to deposit Nb_2_O_5_ thin films, the probability of collisions between atoms is high and this results in the sputtered atoms having higher energies, so they can easily diffuse and the thin films’ structure is more compact. Thus, the refractive index is relatively high. On the contrary, when a lower substrate temperature is used to deposit the Nb_2_O_5_ thin films, the structure becomes less densified and the refractive index would be expected to be lower; therefore, the results of the experiments are as expected. The refractive index values of the Nb_2_O_5_ thin films investigated increased from 2.14 to 2.34 when the O_2_ ratio was 10% and increased from 2.18 to 2.44 when the O_2_ ratio was 20%. When the deposition temperature was RT and 200 °C, the refractive index values of the Nb_2_O_5_ thin films were lower than the values revealed in Reference [19], which changed from 2.23 to 2.28. In the past, there was no research using both the deposition temperature and O_2_ ratio (or they only used the O_2_ ratio) as the deposition parameters. We believe that the high refractive index values in this study are caused by the use of O_2_ during the deposition process, and this will compensate for the oxygen defects in the Nb_2_O_5_ thin films.

**Figure 7:**
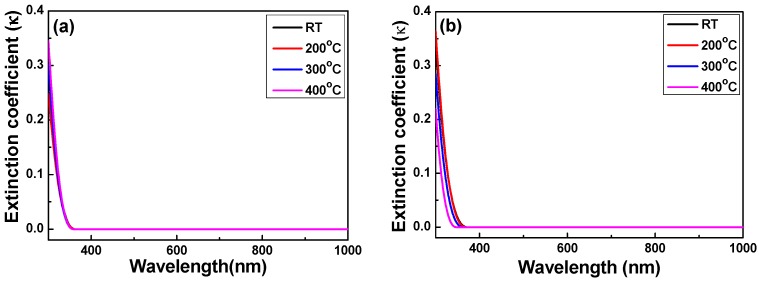
Measured extinction coefficient of Nb_2_O_5_ thin films as a function of deposition temperature and O_2_ ratio. (**a**) O_2_ ratio 10% and (**b**) O_2_ ratio 20%, respectively.

Forouhi and Bloomer deduced dispersion equations for the refractive index, *n*, and the extinction coefficient, *k* [7,29]. The refractive index (*n*) and extinction coefficient (*k*) are related to the interaction between a material and incident light, and are associated with refraction and absorption. Those descriptions suggest again that the properties of the extinction coefficient of Nb_2_O_5_ thin films are very complicated. Figure 7 shows the extinction coefficient of Nb_2_O_5_ thin films as a function of the deposition temperature and the O_2_ ratio in a spectrum range of 350–1000 nm. As the light wavelength changed, the extinction coefficient linearly decreased from ~0.35 when the light wavelength was 350 nm to smaller than 10^−4^ when light wavelength was longer than the 361 nm. When the O_2_ ratio was 10% and the deposition temperature was RT, 200, 300 and 400 °C, the extinction coefficient was 4.05 × 10^−5^, 9.40 × 10^−5^, 4.82 × 10^−5^, and 2.75 × 10^−5^ when the light wavelength was 361, 360, 358 and 357 nm, and the extinction coefficient was zero when the light wavelength was longer than 362, 361, 358 and 357 nm; when the O_2_ ratio was 20% and the deposition temperature was RT, 200 °C, 300 °C, and 400 °C, the extinction coefficient was 2.75 × 10^−5^, 9.81 × 10^−5^, 1.68 × 10^−5^ and 6.89 × 10^−5^ when the light wavelength was 370, 367, 361 and 345 nm, and the extinction coefficient was zero when the light wavelength was longer than 370, 368, 361 and 346 nm, respectively. The extinction coefficient apparently decreases as the deposition temperature increases and it has no apparent trend as the O_2_ ratio is changed. The results in Figure 5 suggest that Nb_2_O_5_ thin films are stable dielectric films with a low extinction coefficient in the range of visible light.

## 4. Conclusions

When the O_2_ ratio was 10% or 20%, the average crystallite sizes increased with the increasing deposition temperature. When the deposition temperature was increased from RT to 400 °C and the O_2_ ratio was 10%, the average total transmittance had no apparent change, the maximum transmittance ratio increased, and the light wavelength to reveal the maximum transmittance ratio was shifted to a higher value (red-shift). When the O_2_ ratio was 20%, the average total transmittance decreased with the increasing deposition temperature. The values of the optical band gap of the Nb_2_O_5_ thin films decreased with the increasing deposition temperature, and the quantum-size effect was the reason causing those results. When the O_2_ ratio was 10% (20%), the refractive index of the Nb_2_O_5_ thin films decreased from 3.14 (3.48) to around 2.13 (2.16) when light wavelength was longer than 450 nm and then the refractive index had a stable value when the light wavelength was longer. The refractive index values of the Nb_2_O_5_ thin films at 550 nm increased from 2.14 (2.18) to 2.34 (2.44) when the O_2_ ratio was 10% (20%) and the refractive index values also increased with the increase of the deposition temperature. We believe that the high refractive index values in this study are caused by the use of O_2_ during the deposition process, and this will compensate for the oxygen defects in the Nb_2_O_5_ thin films. The refractive index at the same light wavelength increased as the deposition temperature was increased from RT to 200 °C and slightly increased as the deposition temperature was further increased. When the O_2_ ratio was 10% (20%) and the deposition temperature was RT, 200, 300 and 400 °C, the extinction coefficient was 4.05 × 10^−5^ (2.75 × 10^−5^), 9.40 × 10^−5^ (9.81 × 10^−5^), 4.82 × 10^−5^ (1.68 × 10^−5^), and 2.75 × 10^−5^ (6.89 × 10^−5^) when the light wavelength was 361 nm (370 nm), 360 nm (368 nm), 358 nm (361 nm), and 357 nm (346 nm), and the extinction coefficient was zero when the light wavelength was longer than 362 nm (370 nm), 361 nm (367 nm), 358 nm (361 nm), and 357 nm (345 nm), respectively. The extinction coefficient apparently decreases as the deposition temperature increases and it has no apparent trend as the O_2_ ratio is changed.

## Figures and Tables

**Table 1:** Average crystallite sizes, variation of crystallite sizes, average thicknesses, and variation of thicknesses of Nb_2_O_5_ thin films.

Atmosphere	Temperature	Average Crystallite Sizes (nm)	Variation of Crystallite Sizes (nm)	Average Thicknesses (nm)	Variation of Thicknesses (nm)
10% O_2_	RT	12.3	10.2–13.9	72.7	70.5–74.7
	200 °C	16.5	14.3–18.5	75.1	73.2–77.5
	300 °C	22.7	19.7–25.9	75.8	73.8–77.3
	400 °C	34.5	31.1–38.6	77.7	75.2–79.6
20% O_2_	RT	11.2	9.5–13.3	47.2	45.8–48.7
	200 °C	15.1	12.5–18.1	47.5	45.7–49.2
	300 °C	21.4	19.4–23.8	47.9	46.1–49.3
	400 °C	32.5	29.7–36.9	46.4	44.9–47.8

**Table 2:** Average total transmittances, maximum transmittance ratio, and light wavelength of maximum transmittance ratio of Nb_2_O_5_ thin films.

Atmosphere	Temperature	Average Total Transmittance	Maximum Transmittance Ratio	Light Wavelength (nm)
10% O_2_	RT	74.42%	85.73%	361
	200 °C	74.15%	90.78%	382
	300 °C	73.87%	91.06%	387
	400 °C	75.17%	91.54%	402
20% O_2_	RT	73.82%	-	-
	200 °C	69.90%	-	-
	300 °C	68.42%	-	-
	400 °C	64.72%	-	-

**Table 3:** Average reflectance, minimum reflectance, and light wavelength to reveal the minimum reflectance of Nb_2_O_5_ thin films.

Atmosphere	Temperature	Average Reflectance	Minimum Reflectance	Light Wavelength (nm)
10% O_2_	RT	26.0%	8.17%	341
	200 °C	26.3%	8.12%	375
	300 °C	26.6%	8.27%	389
	400 °C	25.3%	8.13%	402
20% O_2_	RT	26.7%	14.5%	300
	200 °C	30.6%	13.7%	309
	300 °C	32.0%	12.9%	311
	400 °C	33.7%	9.90%	316

## References

[1] Baltes M., Kytokivi A., Weckhuysen B.M., Schoonheydt R.A., van der Voort P., Vansant E.F. (2001). Supported tantalum oxide and supported vanadia-tantala mixed oxides: Structural characterization and surface properties. J. Phys. Chem. B.

[2] Rani R.A., Zoolfakar A.S., O’Mullane A.P., Austin M.W., Kalantar-zadeh K. (2014). Thin films and nanostructures of niobium pentoxide: Fundamental properties, synthesis methods and applications. J. Mater. Chem. A.

[3] Szymanowski H., Zabeida O., Klemberg-Sapieha J.E., Martinu L. (2005). Optical properties and microstructure of plasma deposited Ta_2_O_5_ and Nb_2_O_5_ films. J. Vac. Sci. Technol..

[4] Yao D.D., Rani R.A., O’Mullane A.P., Kalantar-zadeh K., Ou J.Z. (2013). High performance electrochromic devices based on anodized nanoporous Nb_2_O_5_. J. Phys. Chem. C.

[5] Pollard K.D., Puddephatt R.J. (1999). Chemical vapor deposition of tantalum oxide from tetraethoxo (β-diketonato) tantalum(V) complexes. Chem. Mater..

[6] Mazur M., Szymańska M., Kaczmarek D., Kalisz M., Wojcieszak D., Domaradzki J., Placido F. (2014). Determination of optical and mechanical properties of Nb_2_O_5_ thin films for solar cells application. Appl. Surf. Sci..

[7] Forouhi A.R., Bloomer I. (1988). Optical properties of crystalline semiconductors and dielectrics. Phys. Rev. B.

[8] Rani R.A., Zoolfakar A.S., Subbiah J., Ou J.Z., Kalantar-zadeh K. (2014). Highly ordered anodized Nb_2_O_5_ nanochannels for dye-sensitized solar cells. Electrochem. Commun..

[9] Moghala J., Reidb S., Hagerty L., Gardener M., Wakefield G. (2013). Development of single layer nanoparticle anti-reflection coating for polymer substrates. Thin Solid Films.

[10] Yu Y.Y., Chien W.C., Lin J.M., Yu H.H. (2011). High transparent polyimide/titania multi-layer anti-reflective hybrid films. Thin Solid Films.

[11] Chen Y.C., Yang C.F., Hsueh E.Y. (2010). The application of AZOY transparent conductive oxide film in multifilm-coated polycarbonate optical glasses. J. Electrochem. Soc..

[12] Xiao X., Dong G., Xu C., He H., Qi H., Fan Z., Shao J. (2008). Structure and optical properties of Nb_2_O_5_ sculptured thin films by glancing angle deposition. Appl. Surf. Sci..

[13] Agarwal G., Reddy G.B. (2005). Study of surface morphology and optical properties of Nb_2_O_5_ thin films with annealing. J. Mater. Sci. Mater. Electron..

[14] Georgiev R., Georgieva B., Vasileva M., Ivanov P., Babeva T. (2015). Optical properties of sol-gel Nb_2_O_5_ films with tunable porosity for sensing applications. Adv. Condens. Matter Phys..

[15] Lazarova K., Georgieva B., Spasova M., Babeva T. (2014). Preparation and characterization of mesoporous Nb_2_O_5_ films for sensing applications. J. Phys. Conf. Ser..

[16] Coşkun Ö.D., Demirel S. (2013). The optical and structural properties of amorphous Nb_2_O_5_ thin films. Appl. Surf. Sci..

[17] Lazarova K., Vasileva M., Marinov G., Babeva T. (2014). Optical characterization of sol–gel derived Nb_2_O_5_ thin films. Opt. Laser Technol..

[18] Jose R., Thavasi V., Ramakrishna S. (2009). Metal oxides for dye-sensitized solar cells. J. Am. Ceram. Soc..

[19] Lai F., Lin L., Huang Z., Gai R., Qu Y. (2006). Effect of thickness on the structure, morphology and optical properties of sputter deposited Nb_2_O_5_ films. Appl. Surf. Sci..

[20] Lai F., Li M., Chen K., Wang H., Song Y., Jiang Y. (2005). Substrate temperature effect on the refractive index and a two-step film method to detect small inhomogeneities in optical films. Appl. Opt..

[21] Tauc J., Grigorovici R., Vancu A. (1966). Optical properties and electronic structure of amorphous germanium. Phys. Status Solidi B.

[22] Krishna M.G., Bhattacharya A.K. (1999). Thickness and oxygen pressure dependent optical properties of niobium oxide thin films. Int. J. Mod. Phys. B.

[23] Kurmaev E.Z., Moewes A., Bureev O.G., Nekrasov I.A., Cherkashenko V.M., Korotin M.A., Ederer D.L. (2002). Electronic structure of niobium oxides. J. Alloy. Compd..

[24] Wang F.H., Yang C.F., Liou J.C., Chen I.C. (2014). Effects of hydrogen on the optical and electrical characteristics of the sputter-deposited Al_2_O_3_-doped ZnO thin films. J. Nanomater..

[25] Burstein E. (1954). Anomalous optical absorption limit in InSb. Phys. Rev..

[26] Hamberg I., Granqvist C.G., Berggren K.F., Sernelius B.E., Engstrom L. (1984). Band-gap widening in heavily Sn-doped In_2_O_3_. Phys. Rev. B.

[27] Brayner R., Verduraz F.B. (2003). Niobium pentoxide prepared by soft chemical routes: Morphology, structure, defects and quantum size effect. Phys. Chem. Chem. Phys..

[28] Lvovsky A.I. (2013). Fresnel equations. Encyclopedia of Optical Engineering.

[29] Forouhi A.R., Bloomer I. (1986). Optical Dispersion relations for amorphous semiconductors and amorphous dielectrics. Phy. Rev. B.

